# Neural Network Based Uncertainty Prediction for Autonomous Vehicle Application

**DOI:** 10.3389/fnbot.2019.00012

**Published:** 2019-05-10

**Authors:** Feihu Zhang, Clara Marina Martinez, Daniel Clarke, Dongpu Cao, Alois Knoll

**Affiliations:** ^1^School of Marine Science and Technology, Northwestern Polytechnical University, Xi'an, China; ^2^Porsche Engineering Services GmbH, Bietigheim-Bissingen, Germany; ^3^Cogsense Technologies Limited, London, United Kingdom; ^4^Mechanical and Mechatronics Engineering, University of Waterloo, Waterloo, ON, Canada; ^5^Department of Informatics, Technical University of Munich, Munich, Germany

**Keywords:** neural network, autonomous driving, uncertainty prediction, localization, odometry

## Abstract

This paper proposes a framework for uncertainty prediction in complex fusion networks, where signals become available sporadically. Assuming there is no information of the sensor characteristics available, a surrogated model of the sensor uncertainty is yielded directly from data through artificial neural networks. The strategy developed is applied to autonomous vehicle localization through odometry sensors (speed and orientation), so as to determine the location uncertainty in the trajectory. The results obtained allow for fusion of autonomous vehicle location measurements, and effective correction of the accumulated odometry error in most scenarios. The neural networks applicability and generalization capacity are proven, evidencing the suitability of the presented methodology for uncertainty estimation in non-linear and intractable processes.

## 1. Introduction

Mobility has become a serious challenge in a society with a gradually aging population and a perpetually increasing traffic. This situation is motivating the automotive industry and governments to invest heavily in highly automatized vehicles toward full autonomy. Nevertheless, the progress in autonomous vehicle integration is being hindered from an engineering perspective, due to limits in vehicle sensing and infrastructure modernization requirements (Ma et al., 2018; Taeihagh and Lim, 2019). Moreover, considerable advances in electronics and control theory, safety and robust autonomous driving can only be achieved in conditions that vehicles are fully aware of the driving scenario (Han et al., 2012; Li et al., 2014).

High quality measurements can be obtained using expensive sensors (Elfring et al., 2016), as installed in the well-known Google Car. This vehicle includes an advanced laser range finder, between other sensors, able to process the environment real time (Poczter and Jankovic, 2014). Nonetheless, these advanced devices are generally associated with elevated cost, and therefore are not feasible for serial production vehicles. An alternative solution is to compensate measurements quality with higher redundancy by installing larger number of low cost devices based on different technologies. As a consequence, the features perception becomes a complex problems where heterogeneous signals need to be registered, transformed into a common level and conveniently combined to guarantee safety (Jiang et al., 2011). This process is known as data fusion and usually involves noisy measurements and highly non-linear transformations.

Data fusion can be executed in either centralized or decentralized architectures. Whilst the first involves a common processor, and decentralized architectures consist of networks where each sensor has its own processing unit (Grime and Durrant-Whyte, 1994; Durrant-Whyte et al., 2001; Garcia-Ligero et al., 2012). On the one hand, centralized architectures need to be re-designed when changes in the sensing units take place, which implies costly and time-consuming development. On the other hand, decentralized solutions are particularly convenient in networks where sensors can be dynamically added and removed from the networks as a result of being sporadically available. Nevertheless, although a plurality of measurements might become accessible in decentralized architectures, fusion requires knowledge of the uncertainty associated to them. Furthermore, measurements are not used directly, but information extracted from them hereby referred to as features, which generally involves non-linear transformations. Consequently, the conversion of sensor noise into feature noise is a complex task that usually involves arduous mathematical derivations and can be intractable in many applications.

Several attempts to uncertainty prediction for inertial measurement units (IMU) have been presented in the literature. These include methodologies to fuse odometric measurements with global positioning system measurements, geographical information systems and laser scanners, between others. Vision systems are used in Park et al. (2012) and the disparity between the image space and the Cartesian space is used to derive the uncertainty mathematical model. Vision based controlled is used by Fu et al. (2018) in a system based on an onboard camera and an IMU. The authors use a non-singleton fuzzy logic controller able to handle high uncertainties. The Kalman filter has been also widely used to deal with noisy measurement and models. The parameters estimation was performed using methods such as random walk, Gaussian-Markov and autoregressive processes (El-Diasty and Pagiatakis, 2008). Extended Kalman filters have been proposed by other authors such as Bry et al. (2012) and Fabrizi et al. (2000), where the noise assumption is taken using Gaussian white noise.

This paper presents a solution to facilitate data fusion in decentralized architectures. The proposed paper enhances our previous system for feature extraction (Martinez et al., 2017), where either the source of information, or the sensor noise is unknown. These networks require an appropriate estimation of the signals uncertainty so as to properly fuse them into an “improved measurement,” rather than worsen the fusion output. The uncertainty allows evaluating the quality of the signals and provides a combined result of higher accuracy where the information retained is maximized.

Despite its importance, in the literature revised sensor noise is either assumed to be known, or fitted with simple Gaussian distributions. Hereby, a methodology directly for uncertainty prediction from raw data is proposed based on Artificial Neural Networks (ANN), assuming no information is prior available about the sensor characteristics. The applicability of this data-driven strategy extends to highly non-linear and even intractable feature transformation, avoiding tedious mathematical derivations. Proof of this is supported by its implementation for autonomous vehicle location through odometry data, obtaining satisfactory results in varied scenarios.

## 2. Problem Definition

Autonomous driving highly depends on the sensor measurement, uncertainty and fusion. Nonetheless, sensor models are not generality provided by sensor manufacturers. This lack of data significantly increases challenges in decentralized architectures, where new sensors can be “plugged & played” within the *ad-hoc* network.

### 2.1. Motivation

The operational limits of the sensor technology condition safety in autonomous driving, as a consequence of their strong dependence on the measurements quality (Zheng and McDonald, 2003; Michalke et al., 2011). The GPS precision limits are a well-known example of the noise effect in systems performance, experienced daily by the general public through of the shelf navigation devices (Schrader et al., 2012). This involves the fusion of pseudo-range GPS signals of vehicles, used to minimize the error produced by uncontrolled sources like satellite clock bias, atmospheric delay, and acquisition noise. Nevertheless, despite the complexity of the error origin, previous studies model noise using Gaussian distributions (Liu et al., 2013).

The main barriers for sensor fusion in application, such as vehicle localization, are found in the uncertainty of sensor technology integrated in each vehicle. This inevitably affects the uncertainty characteristics, and the different nature of the signals to fuse, involving highly non-linear feature transformations (Xu et al., 2014). Furthermore, incorrect uncertainty estimation could reduce the fusion accuracy and produce a security hazard by deteriorating the system performance. Regardless of its importance, most research in this area has limited the error prediction to single vehicle model-based approaches usually using Gaussian distributions, developed either theoretically or empirically. Therefore, more accurate uncertainty estimation would be of great benefit for these applications, and would solve issues that hinder the implementation of the autonomous technology nowadays.

### 2.2. Problem Statement

Odometry measurement for vehicle location is subjected to sensor noise applied to velocity *d* and orientation θ. This uncertainty is extended to the features x and y coordinates, which are calculated from the noisy signals through geometrical transformations (Choi and Huhtala, 2016). Consequently, the vehicle location error is accordingly described by non-linear mathematical equations and accumulates along the path with every sampling time. The absence of information about the features uncertainty, x and y covariance, prevents from fusing odometry data with additional measurements that might become available along the trajectory. By means for this, the estimation of the location uncertainty is of great interest to efficiently correct the accumulated error (Zhang et al., 2013). Hereby a feature noise estimator is obtained from data with independence of the sensor characteristics, and complexity of the feature transformation. This solution allows for sensor noise prediction, avoids the use of complex mathematical formulations and facilitates sensor fusion under any use case.

### 2.3. Data-Driven Modeling

Feature transformations are often difficult to derive in mathematical terms, and calculations that are usually time consuming and occasionally intractable. Nevertheless, vehicle trajectory data collection under real-life conditions is generally possible using limited resources. These evidences support the use of data-driven algorithms, methods that can efficiently manage big data and yield insightful conclusions from unknown complex processes (McAfee et al., 2012; Hou and Wang, 2013). Various algorithms can be used to derive the so-called surrogate models without requiring actual understanding of the relationship between inputs and outputs. These models are compact, normally inexpensive to evaluate compared to their homologous strictly mathematically derived. Furthermore, they are mathematically tractable and can estimate the process with high-fidelity at least locally to the training set (Gorissen et al., 2010; Koziel et al., 2011). Some examples of surrogate modeling techniques include: polynomial regressions, kernel methods, kriging, support vector machine, Radial Basis Functions (RBF), and Neural Networks (NN) (Jin et al., 2001; Razavi et al., 2012). Each method has different characteristics in terms of operation, complexity, design flexibility and fidelity capability. For instance, support vectors perform particularly well with high dimensional spaces when only scarce training data is available (Forrester and Keane, 2009). Highly non-linear and complex process are better captured using RBF, kriging, and NN, which require determining a specific number of parameters by trial and error. From a high level analysis, the structural limits of RBF are relaxed with kriging, which assumes the model response has stochastic behavior and fits it with a statistical basis. In the kriging method the basis function variance is considered a parameter, providing larger flexibility and resultant increase in training time.

Artificial Neural Networks allow modeling the relationship between inputs and outputs from data. This characteristic, applied to sensor noise, is expected to be able to find the underlying relation between measurement and noise associated to them. Furthermore, NN accept multiple inputs, which can be used to determine additional features affecting the noise and their correlation. With these precedents, NN offer an exceptional framework for implementing and testing the suitability of models generated from data applied to noise magnitude prognosis of sensor measurements. Hereby, NN are selected within the above strategies to exploit their potential for sensor noise estimation, and explore their high level of flexibility owing to their substantial number of defining parameters: network structure, neuron function, number of hidden layers, and number of neurons per layer.

### 2.4. Design for Surrogate Model Development

In terms of number of hidden layers, the criterion applied focuses on the trade-off between accuracy and generalization capabilities. Sensor fusion algorithms will benefit from a guidance to assess the extent of the error covariance of new sensor measurements. This information will allow the system to identify the degree of information present in the new measurement and perform the data fusion accordingly, ensuring the output maximizes the information content. It is therefore acceptable to obtain a guidance value of this error covariance and not highly precise results, reason why the network structure selected for sensor noise estimation is formed by a single hidden layer. This simplified structure might prevent from learning accurate noise behavior as observed in deep learning, but would also facilitate training and avoid noise fitting when applied to noisy sensor signals. Generalization capability is hereby prioritize against results accuracy with the selection of the single hidden layer structure.

The network layers, named input, hidden and output, can be connected through either feedforward (FF) configuration or using a feedback (FB) connection. Layers in FFNNs only receive information from forward layers, whilst in FBNNs any neurons can connect with each other. Consequently, signals in FBNN are repeatedly transformed and lean toward steady state or vibration state. By introducing feedback delays, this structure is also able to capture the relationship between past inputs and current output, influence that is completely ignored in the FF configuration. In the following, both FF and FB configurations are examined as candidates of NN structure so as to determine the most suitable configuration with the support of the training and test results.

Once the network structure is defined, the size of the layers need to be determined. Input and output layers are constrained by the input and output signals selected set, but the hidden layer is a prior a free parameter, closely related with the process complexity. By reason of lack of known mathematical formulation of this particular case, this number has to be determined by trial and error. The optimal size criteria should consider a trade-off between complexity, accuracy, and generalization capability of the neural network candidates. Excessively complex networks not only raise training time, but also increase the risk of over fitting, which would return high accurate results over the training set and poor generalization capacity on new data (Hagan et al., 2014). The growing method is used in this application in order to prevent for over fitting by establishing an initial network with relative small size, and increasing it gradually with special attention over both the training and testing accuracy.

## 3. Training Data Generation And Analysis

**Figure 1:**
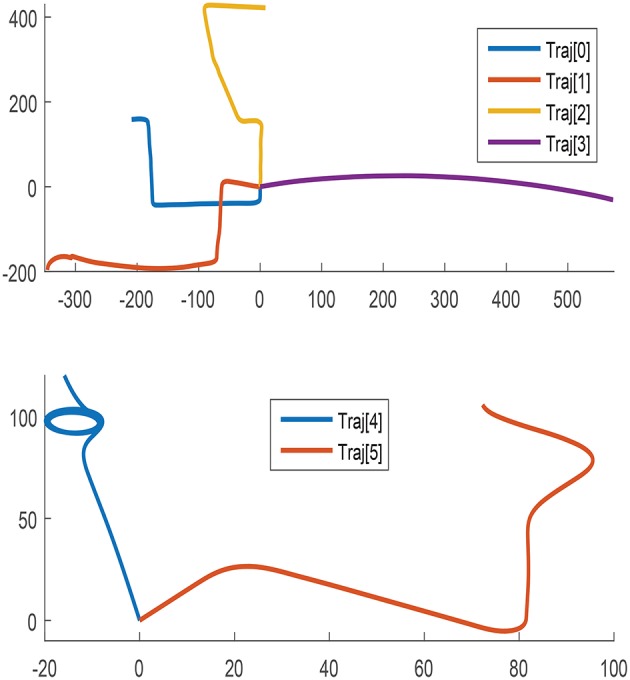
Path followed in all trajectories for training.

To encourage acceptable performance under all possible scenarios, the amount and variability of the training data should ideally account for any conceivable use case. The data selected for training proceeds from six different trajectories that combine disparate direction, length, orientation, and speed as depicted in Figure 1.

### 3.1. Training Data Generation

The trajectories contain highly precise vehicle locations in Cartesian coordinates, and yaw signals, regarded as Ground Truth (GT). The GT signals need to be processed to generate realistic data complying with a real case scenario. Training data is generated assuming the vehicle velocity and orientation, identified herewith with the symbols *d* and θ respectively, are collected with sensors characterized by white noise. For the purpose of the following investigations, the standard deviation values 0.1 and 0.001 are respectively selected for speed and orientation measurements. These values are based on experience and are considered representative of general noise measure in odometry sensors.

• Calculate *d* and θ GT, *d*_*GT*_ and θ_*GT*_, from *x*_*GT*_ and *y*_*GT*_.

• Add white noise artificially to *d*_*GT*_ and θ_*GT*_, through a Monte Carlo (MC) simulation with 1000 iterations. These results in *d* and θ measured (M), *d*_*M*_ and θ_*M*_, and emulates sensor noisy data acquisition.

• Use the inverse equations to calculate *x*_*M*_ and *y*_*M*_ from *d*_*M*_ and θ_*M*_, which in essence is the signal to feature transformation.

• Use the 1000 MC noisy versions of the trajectories to calculate the location error standard deviation (std) in x and y and the location covariance (cov_*xy*_).

**Figure 2:**
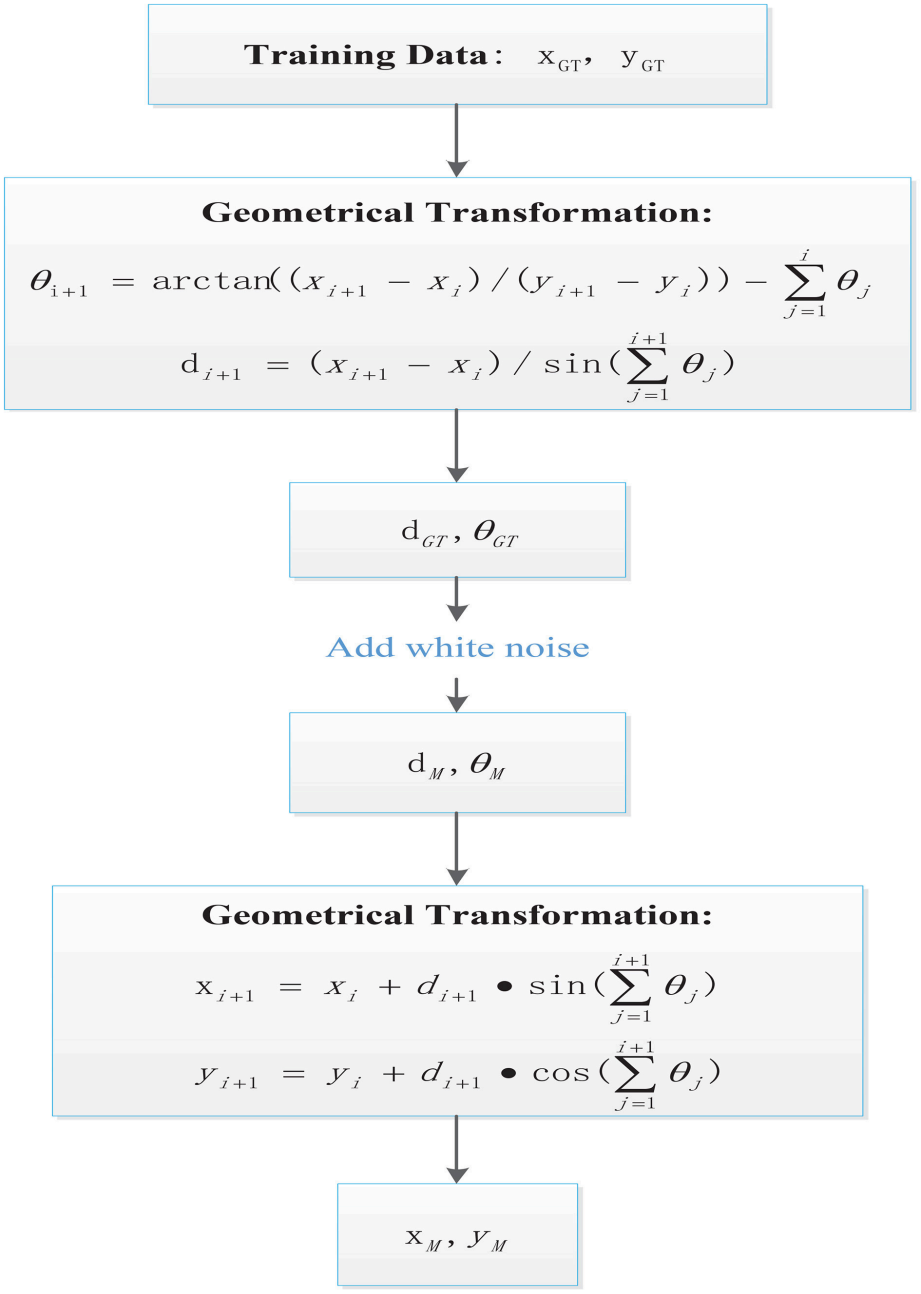
Flow diagram illustrating the ground truth data processing to emulate feature extraction from noisy signals.

Figure 2 illustrates the detailed process in a flow diagram describing the steps and signals obtained from ground truth to measured feature data. As included in the respective steps for geometrical transformations, the signals *d* and θ could be obtained along the sampling steps in a cumulative manner as detailed in following equations (Zhang et al., 2013):

(1) $$\begin{array}{l} x_{n}=\overset{n}{\underset{i=1}{\sum }}d_{i}\cdot \sin\left(\overset{i}{\underset{j=1}{\sum }}\theta_{j}\right) \end{array}$$

(2) $$\begin{array}{l} y_{n}=\overset{n}{\underset{i=1}{\sum }}d_{i}\cdot \cos\left(\overset{i}{\underset{j=1}{\sum }}\theta_{j}\right) \end{array}$$

where *n* refers to the current time step. By combining Equations (1) and (2), the variables of interest can be obtained for every sampling time:

(3) $$\begin{array}{l} \theta_{i+1}=\arctan\left(\left(x_{i+1}-x_{i}\right)/\left(y_{i+1}-y_{i}\right)\right)-\overset{i}{\underset{j=1}{\sum }}\theta_{j} \end{array}$$

(4) $$\begin{array}{l} d_{i+1}=\left(x_{i+1}-x_{i}\right)/\sin\left(\overset{i+1}{\underset{j=1}{\sum }}\theta_{j}\right) \end{array}$$

Noise can be artificially added to the GT results of Equations (3) and (4) by randomly generating numbers with the previously designated standard deviation. The results are regarded as sensor measurements and can be used to obtain the measured features following Equations (1) and (2), implemented as:

(5) $$\begin{array}{l} x_{i+1}=x_{i}+d_{i+1}\cdot \sin\left(\overset{i+1}{\underset{j=1}{\sum }}\theta_{j}\right) \end{array}$$

(6) $$\begin{array}{l} y_{i+1}=y_{i}+d_{i+1}\cdot \cos\left(\overset{i+1}{\underset{j=1}{\sum }}\theta_{j}\right) \end{array}$$

The ground truth original data and measured features can be compared to determine the uncertainty over the vehicle location at every point of the trajectories. This is defined by the standard deviation of the feature estimation error:

(7) $$\begin{array}{l} \sigma_{x}=\sqrt{\frac{1}{N}\overset{N}{\underset{i=1}{\sum }}\left(ex_{i}-\mu_{x}\right)} \end{array}$$

where *N* corresponds to the number of MC iterations of the same trajectory. The estimation error and the mean estimation error can be calculated as follows:

(8) $$\begin{array}{l} ex=x_{GT}-x_{M} \end{array}$$

(9) $$\begin{array}{l} \mu_{x}=\frac{1}{N}\overset{N}{\underset{i=1}{\sum }}ex_{i} \end{array}$$

Similarly, the equations can be applied to y coordinate to obtain *ey*, μ_*y*_, σ_*y*_. Finally, the covariance of the errors in x and y is obtained by:

(10) $$\begin{array}{l} {\text{cov}}_{xy}=\frac{1}{N}\overset{N}{\underset{i=1}{\sum }}\left(x_{i}-\mu_{x}\right)\left(y_{i}-\mu_{y}\right) \end{array}$$

The uncertainty is defined as σ_*x*_, σ_*y*_, and cov_*xy*_. Mean error in x and y bias the location measurement, but are not considered as estimation targets in this particular study. The previous transformation provides information of the uncertainty of the vehicle location, when measured through noisy velocity and orientation sensors subjected to a specific level of white noise. This measurement allows evaluating the quality of the current location through odometry, and therefore to which extent this measure should contribute into a sensor fusion framework when compared to other sources.

**Figure 3:**
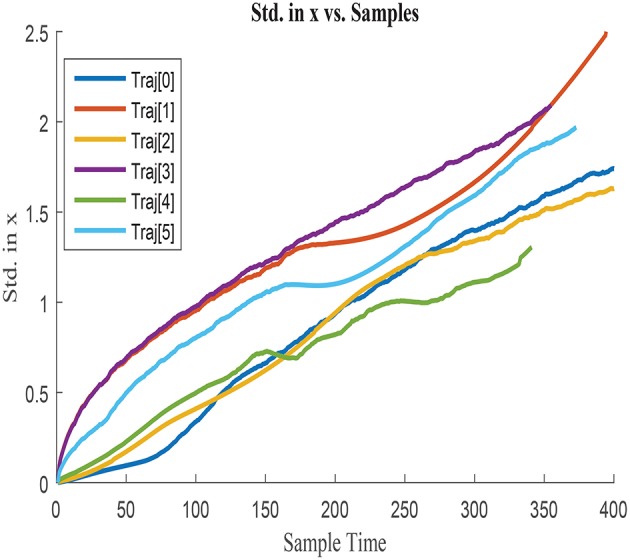
Cumulative error standard deviation in *x*, σ_*x*_ in all training trajectories w.r.t.total steps.

**Figure 4:**
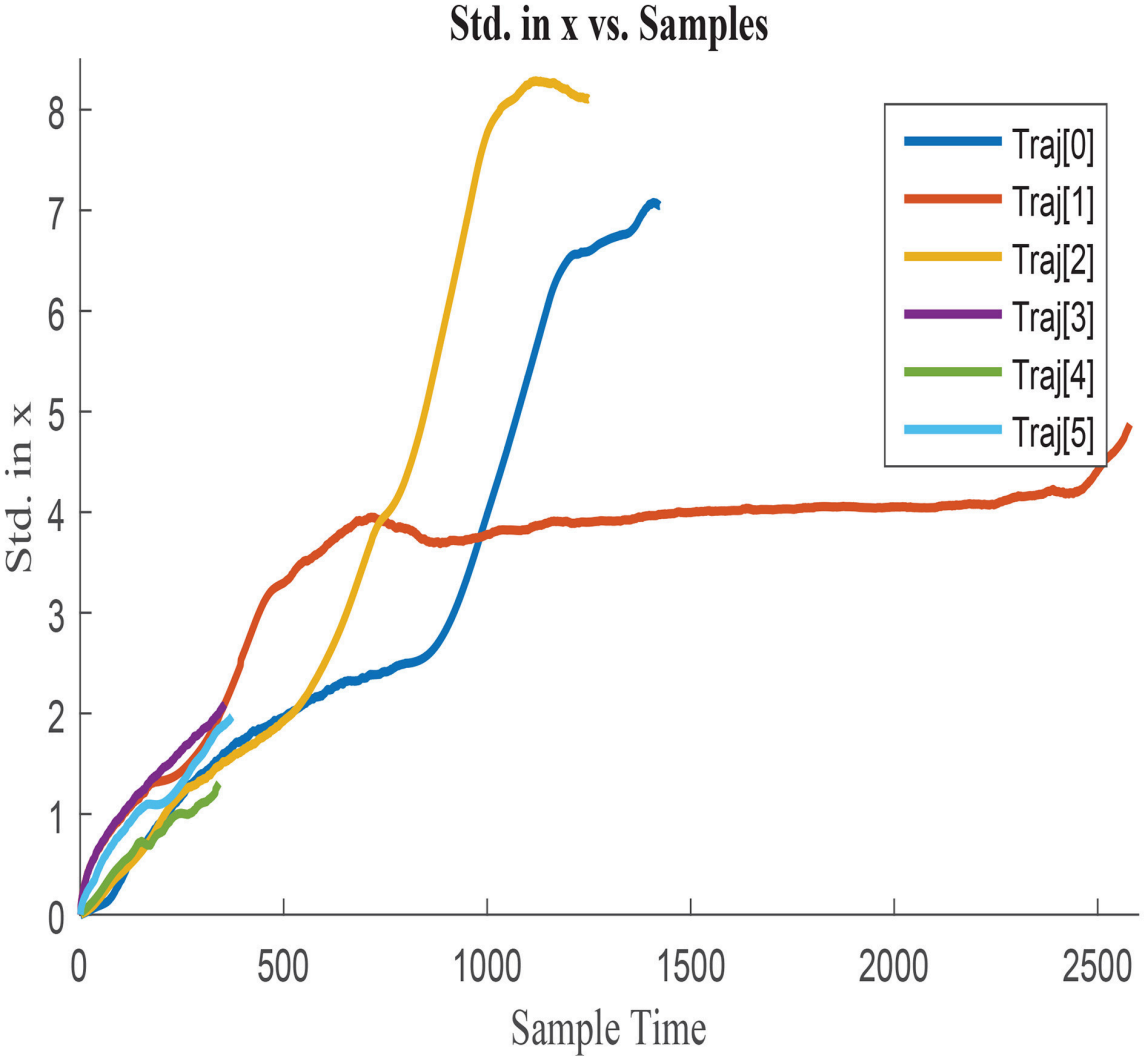
Zoom in cumulative error standard deviation in *x* with shorter duration.

σ_*x*_ obtained for each of the trajectories is illustrated in Figures 3, 4, by assuming the vehicle is perfectly located at the initial point. Figure 3 represents σ_*x*_ growth along the entire trajectories until the end point of the longest one, whilst Figure 4 illustrates a zoom in the σ_*x*_ to better visualize the uncertainty accumulated in shorter paths. σ_*x*_ always presents and increasing trend due to the cumulative characteristics of the error in the vehicle location. Nonetheless, this tendency of accumulation differs between trajectories, which suggests that the shape of the trajectory and the characteristics of the displacement affect the uncertainty growth. The curves are therefore dissimilar between trajectories and presumably influenced by variables such as Δ*x*, Δ*y*, and Δ*yaw*. Analogous behavior is observed when analyzing σ_*y*_ and cov_*xy*_, which agrees with the previous assumption.

**Figure 5:**
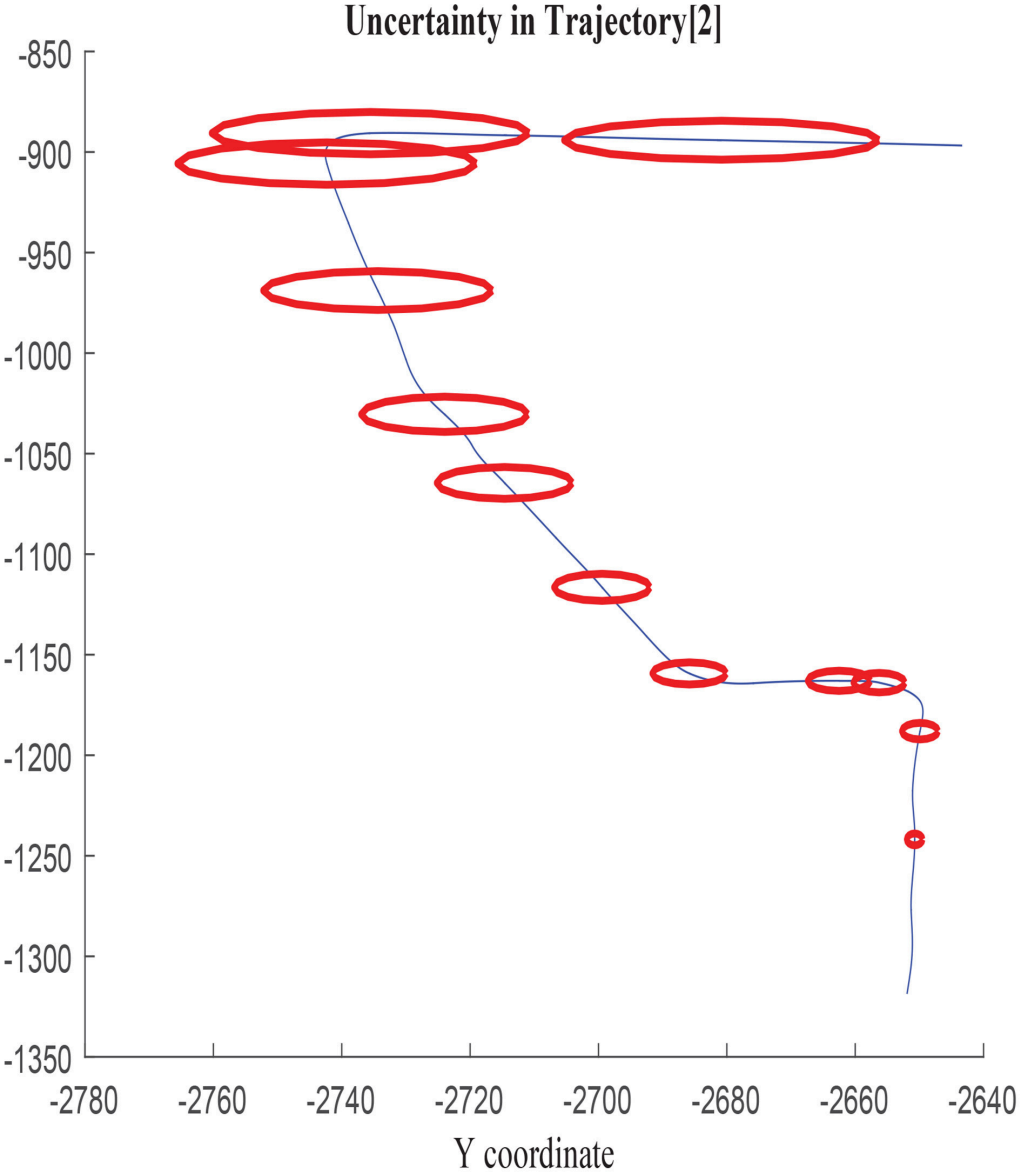
Error ellipses in σ_*x*_, σ_*y*_, and cov_*xy*_ in trajectory 2.

**Figure 6:**
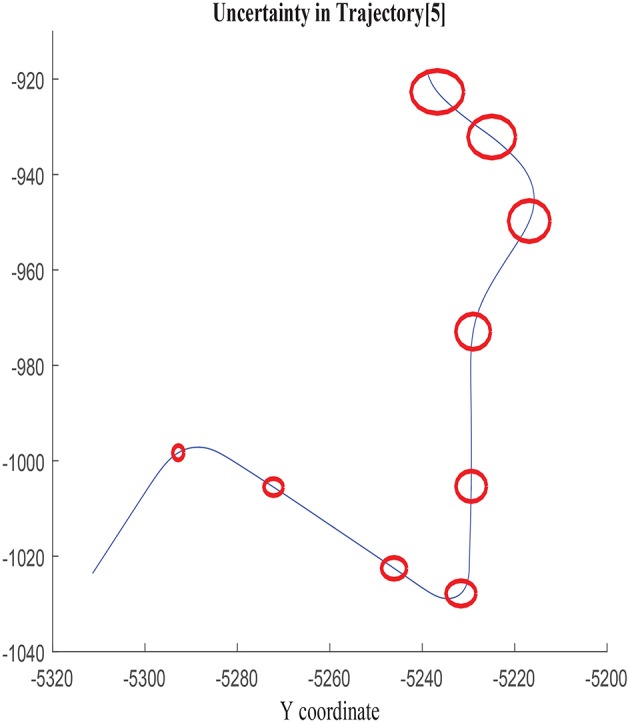
Error ellipses in σ_*x*_, σ_*y*_, and cov_*xy*_ in trajectory 5.

The growth of the combined uncertainty in all directions σ_*x*_, σ_*y*_, and cov_*xy*_ is illustrated in Figures 5, 6, which is represented via ellipses that increase in area as the error accumulates. A first visual examination allows identifying how larger increments in a specific direction affect to the growth of the uncertainty differently, which also allows drawing a priori hypothesis of the variables closely related. In the following, the study concentrates on σ_*x*_, although the results and conclusions are expected to be dimension agnostic and applicable to both σ_*y*_ and cov_*xy*_.

### 3.2. Training Data Analysis

As aforementioned, one of the remaining design parameters of the NN is the number of inputs. Ideally, the input variables should contain the maximum number influencing factors over the target to estimate, but its scope should be constraint to prevent from excessive training time and network complexity and overfitting. The optimal input selection should aim to gather maximum relevant information for the prediction and minimum non-relevant data. Non-useful information would increase the model complexity, and might introduce misleading data that deteriorate the generalization capability.

Inputs can be selected using common sense in easily interpretable applications, albeit there are alternative correlation analysis able to evaluate numerically their level of dependence with respect to the target (Sudheer and Ramasastri, 2002). In addition to a prior evaluation of inputs and outputs, NNs can be themselves used for signals selection. In simple network structures, the importance of each signal can be identified by looking into the magnitude of the weights that connect them to the successive layers. A formal analysis of the signals weights is included in Giordano et al. (2014), where a criteria for input selection is derived mathematically and tested.

As a result of the noisy characteristics of the variables used in this particular application, complex signal evaluation is not considered of interest. Instead, the procedure follows inputs selection, by consisting the combination of a correlation analysis with the training and testing results interpretation of various input candidates. First, the signals linear correlation is studied calculating the Pearson correlation coefficient with respect to the output. The high sampling rate used compared to the input candidates' variability, allows assuming no time lag exists between inputs and outputs, simplifying the evaluation.

Although immediate effect of inputs over the outputs is impracticable, the study of the signals' variability with respect to delayed ones suggests this assumption is acceptable (Maier and Dandy, 1997). The output of the correlation provides useful information to select several input candidates, which are later tested at a second stage to conclude into the most suitable option. The best candidate is assessed in terms of training and testing results in an iterative process.

### 3.3. Inputs vs. Output Correlation

The signals available to use as inputs are: *x, y, yaw*, θ, *d*. The use of values such as real *x* and *y* directly impairs generalization, as the network would learn from training trajectories characterized by specific absolute location points. Moreover, the incremental tendency of the uncertainty suggests additive behavior happening in every trajectory with independence from the initial and relative vehicle location. This reasoning supports the use of signals increments between sampling steps, rather than absolute values with respect to a pre-defined reference system.

Another hypothesis that can be reasonably stated is the effect of the direction of the displacement over the uncertainty growth; that is, whether variables increments or absolute variables increments are suitable for the input set. This conjuncture would be resolved when determining the effect of the inputs increments sign on the uncertainty accumulation. It is sensible to assume that the features uncertainty is affected by the actual magnitude of the displacement with independence on the direction; the uncertainty should not be affected by the reference system. Consequently, it could be deduced that the sign omission would avoid needless information to be fed into the NN, and therefore would encourage the generalization capacity.

All previous hypotheses are considered to determine the signal candidates to evaluate in the correlation analysis. Table 1 contains the Pearson correlation coefficients between input candidates and output in all training trajectories. The formula used is the base line Pearson equation, where *r*, cov, and σ represent respectively correlation coefficient, covariance and variance of the designated signals (Lee Rodgers and Nicewander, 1988).

(11) $$\begin{array}{l} r_{xy}=\text{cov}\left(var_{1},var_{2}\right)/\sigma_{\text{var}1}\sigma_{\text{var}2} \end{array}$$

Table 1 shows high correlation between σ_*x*_ and the signals *yaw, x*, and *y*. *d* appears to have secondary importance, although it proves to be relevant in training trajectories 3 and 4, when compared to 1 and 5 for instance. These differences are associated with the mean value and mean absolute value of the speed, observed to be higher in trajectories 3 and 4. In contrast, θ correlation seems to be negligible.

**Table 1:** Pearson correlation coefficients analysis of input candidate signals, signal increments, and absolute signal increments.

**Signal**							**Signal increment**					**Signal absolute increment**				
**Target**	**Traj**	**x**	**y**	**yaw**	**d**	**θ**	**x**	**y**	**yaw**	**d**	**θ**	**x**	**y**	**yaw**	**d**	**θ**
x std/Increment x std	[0]	–0.8	0.9	–0.7	0.4	0	0.2	0.6	0.1	0	0	0.3	0.6	0.1	0	0.1
	[1]	–0.9	–1	–0.4	0.1	0	−0.1	−0.5	0	–0.1	0	0.1	0.5	0.1	0.2	0.1
	[2]	–0.8	1	–0.7	−0.4	0	−0.5	0.6	0	0.1	0	−0.4	0.6	–0.2	0	−0.1
	[3]	1	–0.5	–1	−0.8	–0.1	−0.2	0.4	0	0	–0.2	−0.2	0.1	0	0	0.2
	[4]	–0.7	0.8	0.9	−0.5	0	−0.2	0.6	–0.3	0.3	0	0.4	0.4	0	0.4	0.1
	[5]	0.9	0.8	0.7	0.2	0	0.3	−0.1	–0.3	–0.1	0	0.5	0	0.4	0.1	0.1

The correlation coefficients change substantially when analyzing variables increments. The weight of Δ*x* and Δ*y* weights reduce, with respect to the original variables and Δ*yaw* becomes practically independent to the output. Δ*y* shows stronger relationship with Δσ_*x*_ when compared to Δ*x*. In contrast, Δ*yaw* are only tangible in trajectories characterized by substantial direction changes, as happens in trajectories 4 and 5. Δ*d* also loses relevance when compared to the absolute variable analysis, and θ influence is barely affected and kept negligible. When focusing on absolute values of the increments, the correlation results produce similar values compared to relative increments, supporting a priori hypothesis over the uncertainty independence with respect to the sign of the movement. A part from the magnitude of the correlation coefficients, the sign can be also interpreted. The broad variety of values and sign within trajectories prevents from selecting a single preferred combination of training signals, reason why several candidates are selected to further pinpoint the suitability.

As a final remark, it is worth highlighting that Δ*y* presents larger correlation with σ_*x*_ than Δ*x*, and vice versa. That is, in trajectories with more movements in x direction, σ_*y*_ grows quicker than σ_*x*_ and similarly, in trajectories with larger displacement in y direction, σ_*x*_ grows quicker than σ_*y*_. This is observed in all trajectories with exception of trajectory 4, where both uncertainties are similar probably due to the followed direction in repeated circles. An explanation of this phenomenon might be found, in the relative amplitude of the actual displacement every sampling and the error magnitude. Whilst the error might be negligible after a large displacement, it could be of the same order of magnitude of short movements, causing larger distortion in the vehicle location. Consequently, large growth of σ_*x*_ could be associated to low Δ*x* instead of being related to Δ*y* as it was initially deduced from the results of the analysis.

### 3.4. Delayed Signals Correlation

Inputs to output correlation analysis is complemented with the signals delay study, also evaluated using the Pearson coefficient. The aim of this test is to determine the possible relationship between old inputs and current outputs; that is, the influence of past changes in the vehicle movement and location on the accumulation of the current uncertainty in the vehicle positioning. The first correlation test of delayed signals analyses the relationship between delayed inputs and current output. The steps used are 0, 1, and 2 sampling times. Next, in order to draw a holistic understanding of the signals interrelation, a second correlation test between current input signals and the same ones delayed 1 and 2 sampling steps is also analyzed.

The results of the first test show similar correlation between input and outputs with independence of the delay implemented. Nonetheless, the second test also shows strong correlation between inputs and the respective delayed inputs. Although from the first results it could be considered that the output depends on past input signals, the second analysis discredits this assumption as they could also be due to the high similarity between current and past inputs. Consequently, no conclusive assumptions can be derived from this correlation test.

The results from the second correlation test effectively show that the inputs and delayed version of the inputs are practically identical, and consequently show similar correlation with the output. As previously states, this similarity might be due to the small sampling step implemented with respect to the input signals variability in time. Further investigations should be conducted to arise conclusive answers to the previous hypothesis. Accordingly, additional study with respect to the delay effect of the feedback NN states is considered during training.

## 4. Training Sets Candidates

Hereby, a training set is defined as the union of a specific combination of input signals, obtained from a selected number of training trajectories in an enclosed array used as training data. That is, the training set is defined by the signals used between the candidates previously analyzed in the correlation analysis and the trajectories from which signals are extracted. The training sets can contain data proceeding exclusively from a single trajectory or from the combination of more than one. Furthermore, the same trajectory can be repeated in each set in more than one occasion by implementing different noisy version from the 1000 MC simulations, practice that intents to encourage the response robustness to the presence of noise in the inputs.

### 4.1. Training Sets

The input training sets are designed in terms of number of signals, trajectory characteristics and amount of trajectories used, and always contain noisy data so as to simulate with maximum fidelity real case studies. Table 2 includes the training set candidates carefully designed to determine: the most suitable combination of inputs, optimal network structure and size and data variability requirements.

**Table 2:** Input sets candidates proposed for training and testing including: signals selected, data used, and hypothesis to verify/reject in the training results.

**Set**	**Inputs**	**Data**	**Explanation**
1	Δ*x*_*M*_,Δ*y*_*M*_,Δ*yaw*_*M*_	All trajectories-3 times	Input:relative increment
2	Δ*x*_*M*_,Δ*y*_*M*_,Δ*yaw*_*M*_	Trajectory[0]-10 times	Data:generalization capacity
3	abs(Δ*x*_*M*_,Δ*y*_*M*_,Δ*yaw*_*M*_)	Trajectory[0]-10 times	Input:generalization of abs. inc
4	abs(Δ*x*_*M*_,Δ*y*_*M*_,Δ*yaw*_*M*_)	Traj.[1],[4,][5]-10 times	Data:generalization disparate data
5	abs(Δ*x*_*M*_,Δ*y*_*M*_,Δ*yaw*_*M*_)	Traj.[1],[4,][5]-5 times	Data:generalization disparate data
6	abs(Δ*x*_*M*_,Δ*y*_*M*_,Δ*yaw*_*M*_ and Δθ_*M*_)	Traj.[1],[4,][5]-5 times	Input:proof of correction analysis
7	abs(Δ*x*_*M*_,Δ*y*_*M*_ and Δθ_*M*_)	Traj.[1],[4,][5]-5 times	Input:proof of correction analysis
8	abs(Δ*y*_*M*_ and Δθ_*M*_)	Traj.[1],[4,][5]-5 times	Input:proof of correction analysis
9	abs(Δ*x*_*M*_,Δ*y*_*M*_ and Δ*yaw*_*M*_)	All trajectories-3 times	Additional testing
10	abs(Δ*y*_*M*_ and Δθ_*M*_)	All trajectories-3 times	Additional testing

The second column in Table 2 specifies the input signals used in each set, where abs and Δ indicates absolute value and signals increment, respectively. The amount of data used in each training set is detailed in the third column, alluding to the variability of trajectories used and amount of MC noisy versions of each trajectory. For instance, set 1 considers all trajectories repeated three times each; including therefore three MC noisy versions of each. The use of larger number of trajectories or specific ones is thoroughly defined, so as to reflect changes in the generalization capability with respect to training data variability. Consequently, by repeating noisy version of the same trajectory, the data variability should be much less than noisy versions of different trajectories.

**Figure 7:**
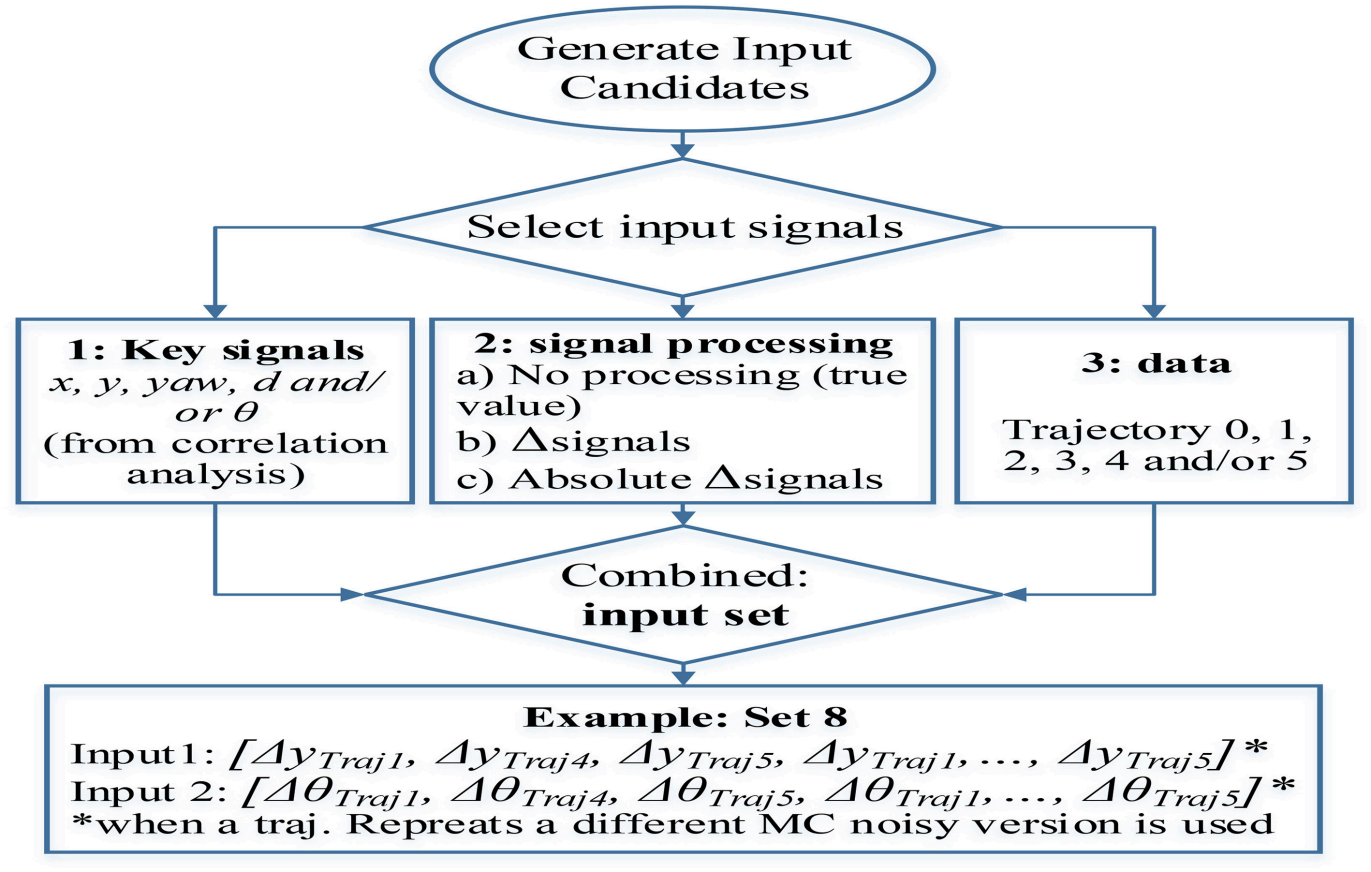
Training sets generation from signals proceeding from the available trajectories including signals selection (from correlation analysis), signal processing (true value, increment, or absolute increment), and training trajectories used (data variability).

The incremental variables specified in Table 2 are calculated, with respect to the constant sampling rate in training sets. As an attempt to encourage generalization, alternative incremental inputs are proposed by using random dynamic sampling within the boundary of 1 to 9 sampling steps. Nonetheless, the networks trained with this data were not able to estimate the target variable, reason why they are neither included in Table 2 nor in the test results. The failure to capture the process could be excused in the data variability and complexity introduced through variable sampling. The networks trained with this data were presumably required to emulate a behavior more complicated than the one described with constant sampling. Consequently, it might be the case that the amount of training data and network size used were not suitable to capture efficiently the underlying process. Figure 7 illustrates a flow diagram that clarifies the design process and characteristics that define a training set. The input selection is partitioned in three stages: selection of the key signals combination, format of the signals preferred (real value, increments, or absolute increments) and trajectories used to extract the data.

### 4.2. Training Candidates

Sets 1 and 2, similarly to 3 and 4, compare the effect of the data variability on the generalization capacity by implementing identical input variables, but using data from different trajectories. Sets 2 and 3 determine the effect of the inputs sign again with respect to generalization, and intend to provide numerical support to proof the independence between uncertainty accumulation and movement direction. Sets 5 to 8 implement identical data, but use different input candidates so as to obtain the optimal signals combination.

## 5. NN Design And Training

The NN candidates are compared in terms of: network size, structural complexity and results output quality. The accuracy of the estimation is evaluated using various error measurements applied to both network output, Δσ_*x*_, and target variable σ_*x*_.

### 5.1. Error Measurements

The results are evaluated by using Root-Mean-Square (RMS) error and relative error measures between the network output and reference data, GT variables. The error measurement include: RMS of σ_*x*_ and relative error of the accumulated uncertainty obtained at the end of the trajectory, σ_*x*_(end). The first assesses the actual performance of the network given the fact that the target variable is the increment of the uncertainty, Δσ_*x*_, and not the cumulative one, σ_*x*_. The second, σ_*x*_ RMS, evaluates the variable of interest and determines the possible predicted error accumulation and the actual one. Finally, the relative error of the final cumulative uncertainty analyzes how well the NN would perform in case a new sensor becomes available at the end of the vehicle path and fusion is required.

These indicators are calculated for each of the trajectories separately, so that it is possible to compare the performance in both, data used for training and data not seen before. It is also worth mentioning that the data used for training in all cases consist of 70% of the total amount that defined the training set, whilst the rest is used for testing and validation during the training process. None of the sets or NN configurations converge during the training process, when using the LR architecture as a consequence of the noisy characteristics of the data used. Nonetheless, this behavior is not necessarily detrimental due to the fact that the generalization capability is preferred to the estimation accuracy of a specific trajectory; it is of importance to avoid noise fitting. Consequently, the networks are trained up to a certain performance value or number of iterations, epochs, and early stopping is used before the training gradient stabilizes. The output to estimate is the relative increment of the error in x std in all cases, Δσ_*x*_.

### 5.2. Training Algorithms

Two algorithms are used for training, Levenberg-Marquardt (LM) and Scaled Conjugate Gradient (SCG). These aim to compensate from deficiencies in terms of robustness, and convergence time of the well-known Error Backpropagation (EBP) and Gauss-Newton algorithms (Moller, 1993; Yu and Wilamowski, 2011). Both differ in the selection of the step size and direction during convergence. Ideally, longer steps should be implemented at early stages and gradually smaller ones should be considered to encourage the result finesse in later stages. Moreover, the error shape might also change, affecting simultaneously to the optimal step direction. SCG implements optimized step size and direction, whilst LM alternates EBP and Gauss-Newton methods depending on the error shape. LM combines the advantages of both strategies taking advantage of the speed convergence of Gauss-Newton with quadratic error, and the robustness of EBP convergence behavior under conditions of non-advantageous for Gauss-Newton.

The training results usually benefit from LM when compared to SCG throughout the test cases. Furthermore, LM presents low μ values, variable that determines the alternation between methods, but it converges neither into Gauss-Newton nor into the steepest descent method.

### 5.3. Training Results: Input Signals Selection

Table 3 summarizes the results obtained after training specific network structures, second column, with the training sets enumerated in the first column. Training set 2 is used to compare FF and LR networks. In all cases, the training with identical set and structure is repeated in more than one occasion, typically up to six times. This practice is recommended due to the possible effect that the random weights initialization can cause over the end solution, which can potentially be trapped into local optima. The results included in Table 3 are taken from the best network obtained after training several candidates. These figures are compared between equal amounts of iterations.

**Table 3:** Training results comparison and analysis in terms of structural and data complexity, training time and performance, and accuracy indexes as convey for error evaluation.

	**Structure**	**Performance**	**Epochs**	**Data Q**	**RMS**	**rms Cmltv**	**End error**
Set 2	100FF	5.99E-06	136	14130	0.059	9	19
Set 2	20LR	9.23E-07	200	14130	0.0447	7.11	9.07
Set 3	20LR	1.07E-06	200	14130	0.0286	4.96	7.46
Set 3	30LR	1.25E-06	82	14130	0.032	4.7	6.6
Set 4	30LR	8.12E-04	67	32900	0.265	119	197
Set 5	30LR	5.92E-05	110	16450	0.22	84.2	67.7
Set 6	30LR	2.60E-05	150	16450	0.24	106.9	88.2
Set 7	30LR	4.50E-05	150	16450	0.27	100.4	80.7
Set 8	30LR	6.15E-05	127	16450	0.18	43.4	30.4
Set 9	40LR	4.67E-06	223	18927	0.0190	2.1	2
Set 10	40LR	4.71E-06	250	18927	0.0220	3.6	4.8
Set 9	30RL	3.56E-06	137	18927	0.0223	3.8	3.9
Set 9	50LR	4.71E-06	300	18927	0.0185	2.6	3.6

Sets 1 and 2 are omitted for brevity, as the conclusion coincides with the analysis of sets 3 and 4. Set 2 is used to implement FF and FB configurations as included in the first two rows of Table 3. The noise filtering capability of FBNN improves the estimation accuracy notably, reason why the LR structure is concluded as the most suitable and successively used in the subsequent training sets.

Six networks trained with set 2 are compared to six networks trained set 3, to determine the effect of sign in the generalization capacity. The best candidate from set 3 shows that the error is 30% lower compared to the best candidate provided by set 2. The results evidence the benefits of reducing non-relevant data in the input set, and corroborate the independence of the location error accumulation with respect to the movement direction.

Sets 3 and 4 evaluate the relationship between generalization and variability of the training sets. The candidates are trained for similar number of epochs using the same structure and size. Networks trained with set 4 are expected to have improved generalization capacity, when compared to the ones trained with set 3, oppositely to the results detailed in rows four and five. These results are not conclusive due to the deficient amount of training time allowed for set 4 network candidates, but show evident differences between networks complexity when trained with each set. Larger data variability would presumably imply also higher network complexity, and therefore longer training time and number of epochs.

Sets 5 to 8 implement the same data variability, but use different combination of input signals. The training is programmed for similar number of epochs in all cases, and therefore the results are expected to benefit simpler training sets. By comparing sets 5 and 7 it can be deduced that the yaw is preferred to θ, and therefore set 6 does not benefit from the additional information. Nevertheless, the result of set 8 do not corroborate this hypothesis, reason why extra training is programmed with sets 9 and 10. These last sets incorporate maximum data variability, and are trained for a larger number of iterations up to a satisfactory performance. As expected from the preliminary results obtained in sets 5, 6, and 7, the combination of inputs used in sets 5 and 9, Δ*x*, Δ*y*, and Δ*yaw*, is superior to the other candidates. These results were already anticipated by looking into the relationship between coordinates x and y and odometry signals *d* and θ, which geometrically dependent through (1) and (2). Consequently, it is reasonable to assume that the combination of any of the previous pairs would not provide extra information to the training set. In contrast, yaw proceeds from a three dimensional displacement incorporating new data that seems to the valuable for uncertainty prognosis.

### 5.4. Delay Effect on the Training Results

The effect of the feedback delay over the estimation accuracy is studied in the LR configuration by implementing: 1 sample delay, 2 samples delay and the combination of both. The results obtained do not vary substantially with incremental delays, which supports the hypothesis of independence between delayed inputs and current outputs. Nevertheless, the output precision benefit in all cases from the noise filtering effect of feedback structures, reason why 1 step delayed is selected.

**Figure 8:**
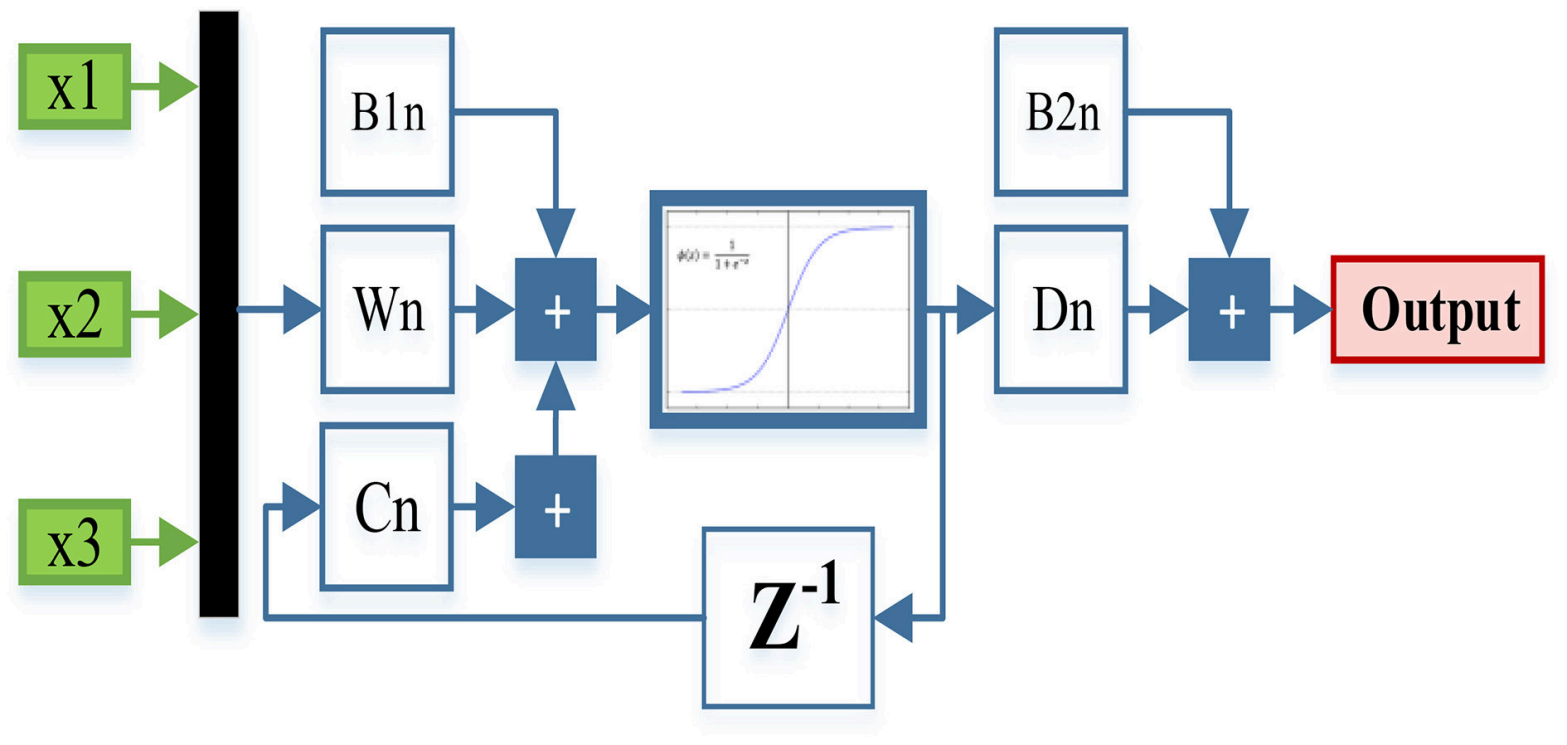
Final structure to find the hidden layer.

Figure 8 presents the structure used to find the optimal hidden layer size, where *n* corresponds to the number of neurons in the hidden layer.

### 5.5. Hidden Layer Optimal Size

**Figure 9:**
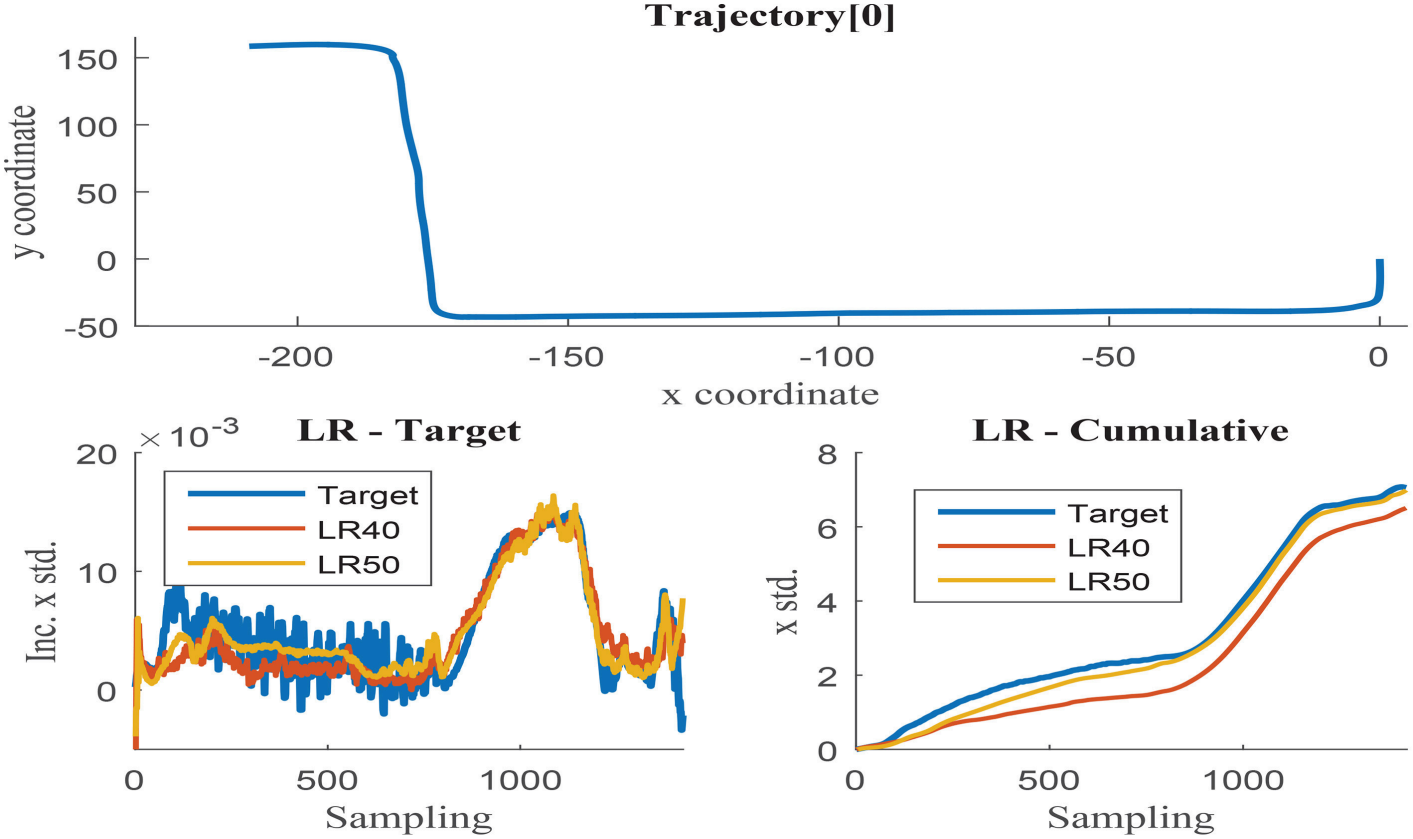
**(Top)** x and y coordinates of trajectory 0; **(Bottom-Left)** Δσ_*x*_ in trajectory 0 with respect to the sensor sampling; **(Bottom-Right)** Δσ_*x*_ in trajectory 0 with respect to the sensor sampling.

**Figure 10:**
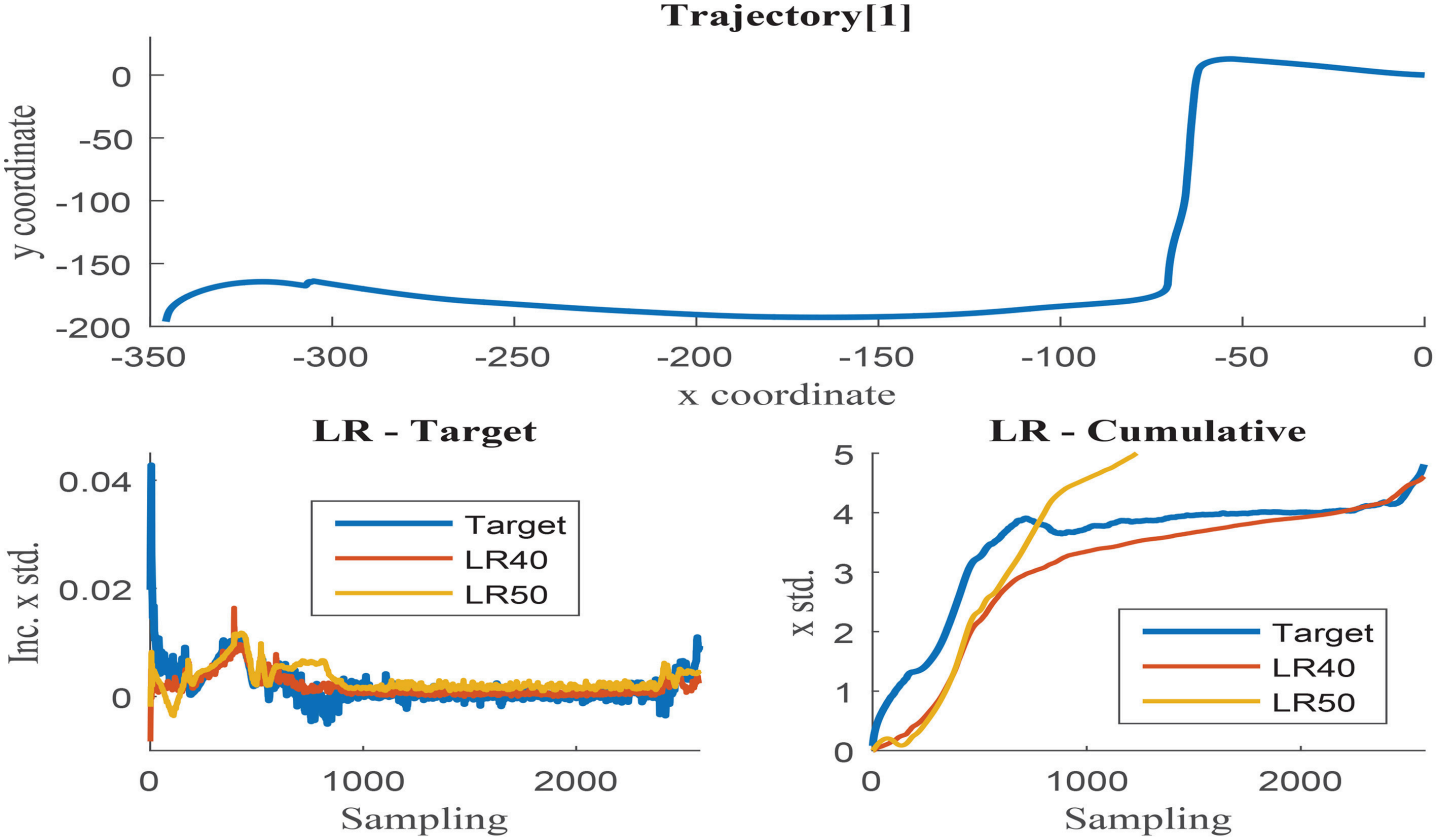
**(Top)** x and y coordinates of trajectory 1; **(Bottom-Left)** Δσ_*x*_ in trajectory 1 with respect to the sensor sampling; **(Bottom-Right)** Δσ_*x*_ in trajectory 1 with respect to the sensor sampling.

Further training with set 9 using 20, 30, 40, and 50 neurons was programmed to determine the optimal hidden layer size. Networks with 20 and 30 neurons presented unacceptable error measures as they were not able to capture the process complexity. Fifty neurons networks were able to accurately estimate the cumulative uncertainty in most of the trajectories, but presented inconsistent behavior in some cases. The estimation results obtained with 40 and 50 neurons networks are visually compared in Figures 9, 10. These illustrations are formed by three graphs, the trajectory shape at the top level and the uncertainty estimation at the low level, including the uncertainty increment of the left and the accumulation on the right.

Figure 9 illustrates trajectory 0, in which the 50-neurons network returns improved results when compared to the 40-neurons networks. Higher number of neurons are able to filter the noisy inputs more effectively, as illustrated in the left graph, and seem to follow better the uncertainty increment, almost matching the cumulative value at the end of the trajectory. The estimation results of the 40-neurons network also match the increments in uncertainty and the shape of the accumulated error, but it is not able to effectively filter the noise. It could be deduced from the previous results that the more the noise is filtered, the better the estimation accuracy obtained. Nevertheless, this is not the case observed when analyzing trajectory 1 as illustrated in Figure 10. Although again 50-neurons networks filter the noise in the uncertainty increments in the left graph, the tendency of the cumulative uncertainty diverges from the target variable causing inconsistent behavior. Oppositely, 40-neurons networks are able to both filter the noise and follow the cumulative uncertainty tendency, returning reasonably accurate results at the end point of the trajectory.

Although 50-neurons network are able to return very accurate results in most of the training trajectories, they show inconsistent behavior at times, which notably diverge from the target. As previously stated, the generalization capability primes in front of the estimation accuracy in the specific application of sensor fusion. Consequently, 40-neurons networks are considered to be the best candidate to model the uncertainty increment, and are therefore considered as reference size in the following tests.

These results agree with the so-called Ockham's Razor principle, which prefers simpler networks structures able to provide acceptable level of accuracy, rather than complex and more accurate ones. The 50-neurons network is able to capture higher non-linear process than the smaller versions. This extra modeling capacity could be either trained to fit the process more accurately, or to capture other processes such as inputs noise, excusing the divergent results depicted in Figure 10. Nonetheless, it is not a suitable network size for the characteristics of the available training data.

## 6. Test Results

Given the fact that the NN has seen 70% of the data used for testing, although different version of the noisy trajectories are used, these still share similar characteristics which might prevent from yielding final conclusions. Furthermore, NNs can be considered as blackbox models whose robustness cannot be tested with conventional methods. In order to further analyze the candidate performance, 28 new trajectories combining straight lines, sharp turning and winding routes along urban areas are used for testing.

### 6.1. Set Candidate 9

Likewise to the methodology followed to process the training trajectories, the test trajectories are converted into noisy features emulating data collection through noisy sensors. Again 1000 MC noisy versions are generated to model the uncertainty accumulation along the path. The estimation performance of the 40-neurons network is tested in all 1000 noisy version of each of the 28 trajectories so as to obtain the average error: RMS of Δσ_*x*_, RMS of σ_*x*_, and end error of the cumulative uncertainty. Although the estimation results are in average satisfactory, the network candidate is not able to fit error accumulation with appropriate accuracy in all test cases. Due to the characteristics of blackbox model of the NN, it is difficult to predict in which case scenarios the network will be able to capture the uncertainty growth.

**Figure 11:**
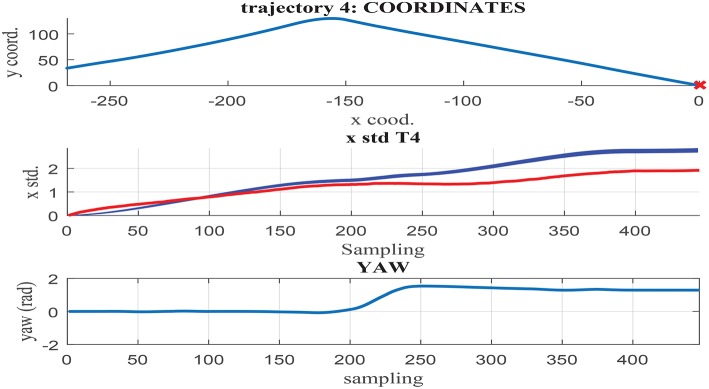
**(Middle)** σ_*x*_ estimation in MC noisy trajectories (blue) respect to path coordinates **(Top)** and yaw **(Bottom)** in trajectory 4.

**Figure 12:**
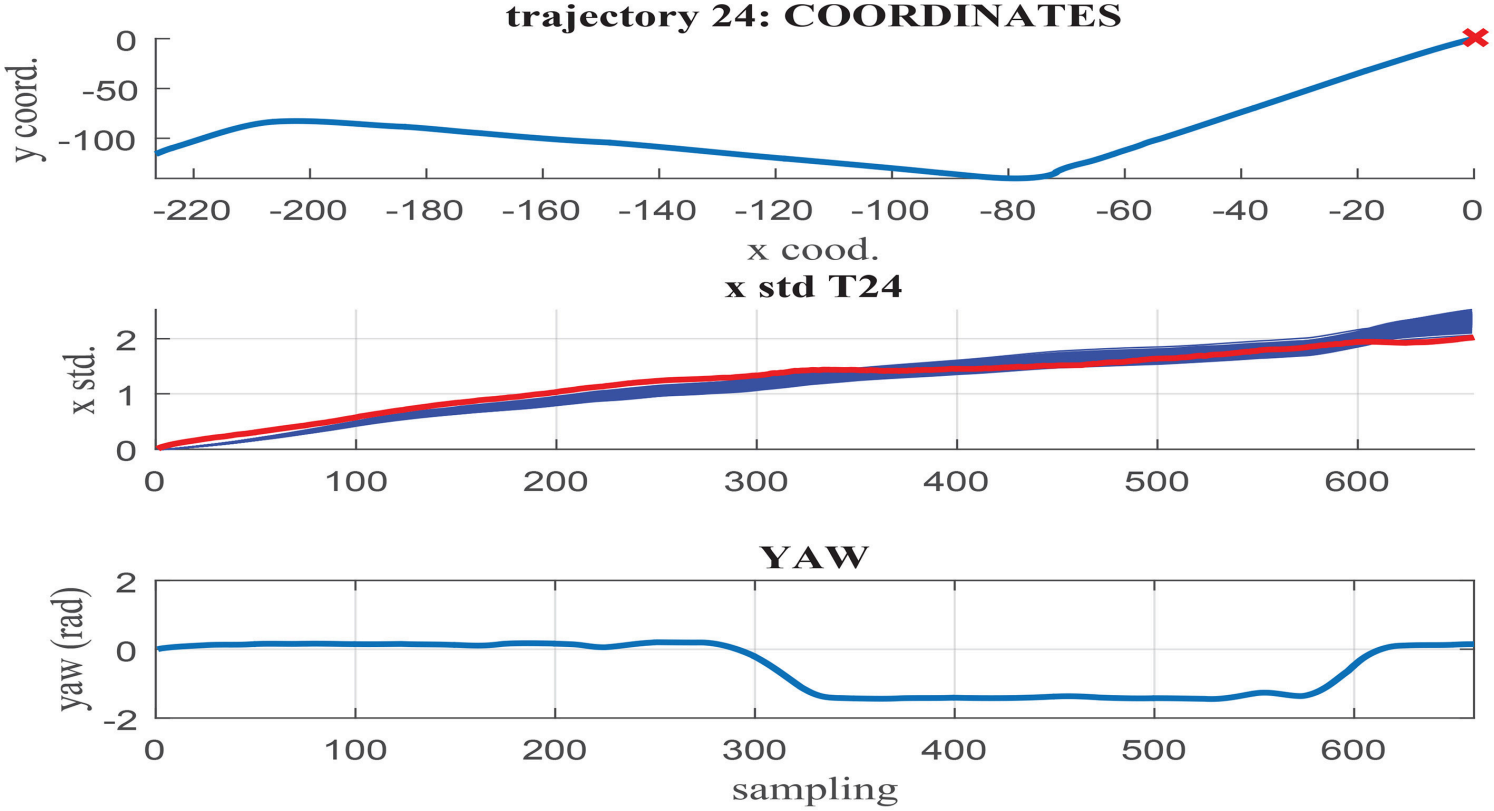
**(Middle)** σ_*x*_ estimation in MC noisy trajectories (blue) respect to path coordinates **(Top)** and yaw **(Bottom)** in trajectory 24.

**Figure 13:**
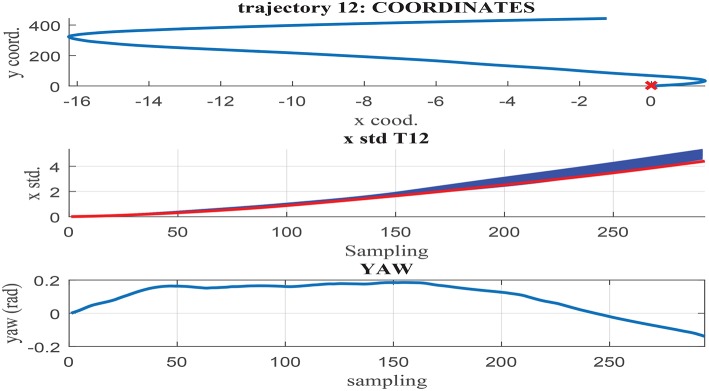
**(Middle)** σ_*x*_ estimation in MC noisy trajectories (blue) respect to path coordinates **(Top)** and yaw **(Bottom)** in trajectory 12.

Figures 11–13 illustrate the results obtained in test trajectories 4, 24, and 12, respectively. These contain three graphs including the trajectory coordinates, the cumulative uncertainty and the yaw signal respectively from top to bottom. The middle graph illustrates in red the targeted cumulative uncertainty and 1000 estimation outputs of the network in blue. Although the cumulated uncertainty is well-captured in trajectories 24 and 12, this is not the case of trajectory 4. These results can be explained through the values of the *yaw* signal in this test scenarios. The first 200 sampling steps are characterized by close-to-zero yaw, followed by a large increment in yaw and a close-to-constant value until the end of the route. When looking into the tendency of the accumulated uncertainty, the shape seems to match the growth only in the last 200 sampling steps. This behavior is observed in some of the testing trajectories, suggesting a consistent response. Furthermore, when assessing the training trajectories, no scenario with large yaw changes is observed. As a consequence of this deficient training data, the network is not able to capture the uncertainty when these conditions take place in the testing trajectories.

In contrast to testing trajectory 4, the trajectories 24 and 12 do not present zero *yaw* values and maintain it mostly constant along the whole path. As a consequence, the network is able to accurately fit the uncertainty accumulation, with accuracy acceptable to allow for sensor fusion at any point of the route. It can be concluded from the previous that the generalization capacity of the network could be potentially improved provided that the training set includes the test cases missing.

### 6.2. Set Candidate 11

The deficiencies of training set 9 data variability are corrected in candidate 11. This implements the same input signals, absolute value of Δ*x*, Δ*y*, and Δ*yaw*, but includes a larger amount of trajectories and therefore a larger number of case scenarios that include possible changes in the *yaw* signal, not previously captured. Whilst set 9 only considers data from the training trajectories 0 to 5, set 11 also includes some of the trajectories previously used for testing; test trajectories 1, 2, 3, 4, 9, 14, 22, and 24. Table 4 contains the details of the sets characteristics, data used, network structure, training and testing results. This table includes the results corresponding to the best network trained with sets 9 and 11. Again, new networks are trained with identical structure and data, and the best is selected to avoid deceiving results caused by random weights initialization. The results included in Table 4 evaluate the networks performance in all testing trajectories, including the ones also used for training in set 11.

**Table 4:** Comparison of training sets 9 and 11 in terms of set characteristics and testing results in trajectories not used for training.

	**Set 9**	**Set 11**
Input signals	abs(Δ*x*,Δ*y*,Δ*yaw*)	abs(Δ*x*,Δ*y*,Δ*yaw*)
Training	Train trajectories	Train trajectories 0-5
		&
		Test trajectories 1,2,3,
		4,9,14,22 and 24
**TRAINING DETAILS**		
NN structure	40 neurons LR	40 neurons LR
No.epochs	223	126
Training	4.67·10^−6^	8.20·10^−6^
Performance		
**AVERAGE TEST RESULTS IN ALL TRAJECTORIES**		
Δσ_*x*_ agv.RMS	0.00469	0.004691
σ_*x*_ agv.RMS	0.4	0.45
avg.end error	0.54	0.52
**AVERAGE TEST RESULTS IN SET 9 TRAINING TRAJECTORIES**		
Δσ_*x*_ agv.RMS	0.00445	0.004518
σ_*x*_ agv.RMS	0.41	0.35
avg.end error	0.52	0.43
**AVERAGE TEST RESULTS IN SET 11 TRAINING TRAJECTORIES**		
Δσ_*x*_ agv.RMS	0.004838	0.004798
σ_*x*_ agv.RMS	0.4	0.51
avg.end error	0.55	0.58

In average, the prediction accuracy of the increment in the uncertainty, Δσ_*x*_, is identical in both cases when analyzing the average accuracy in all test trajectories. This result suggest that the 40 neurons LRNN has reached a performance limit with set 9 and does not admit the further complexity provided in set 11. Moreover, set 9 presents better accuracy when analyzing the average RMS error in σ_*x*_ and worst results when comparing the end error.

When looking into the trajectories used to train set 11, as expected the results provided by the network trained with set 9 are worse. In this case set 11 has the advantage of having implemented 70% of those trajectories during training. Nevertheless, the accuracy of the network trained with set 11 is worse when looking exclusively to the trajectories not used in any of the sets. This result could be explained considering the loss of generalization capability, when the training data complexity overcomes the non-linear capacity of the network structure.

**Figure 14:**
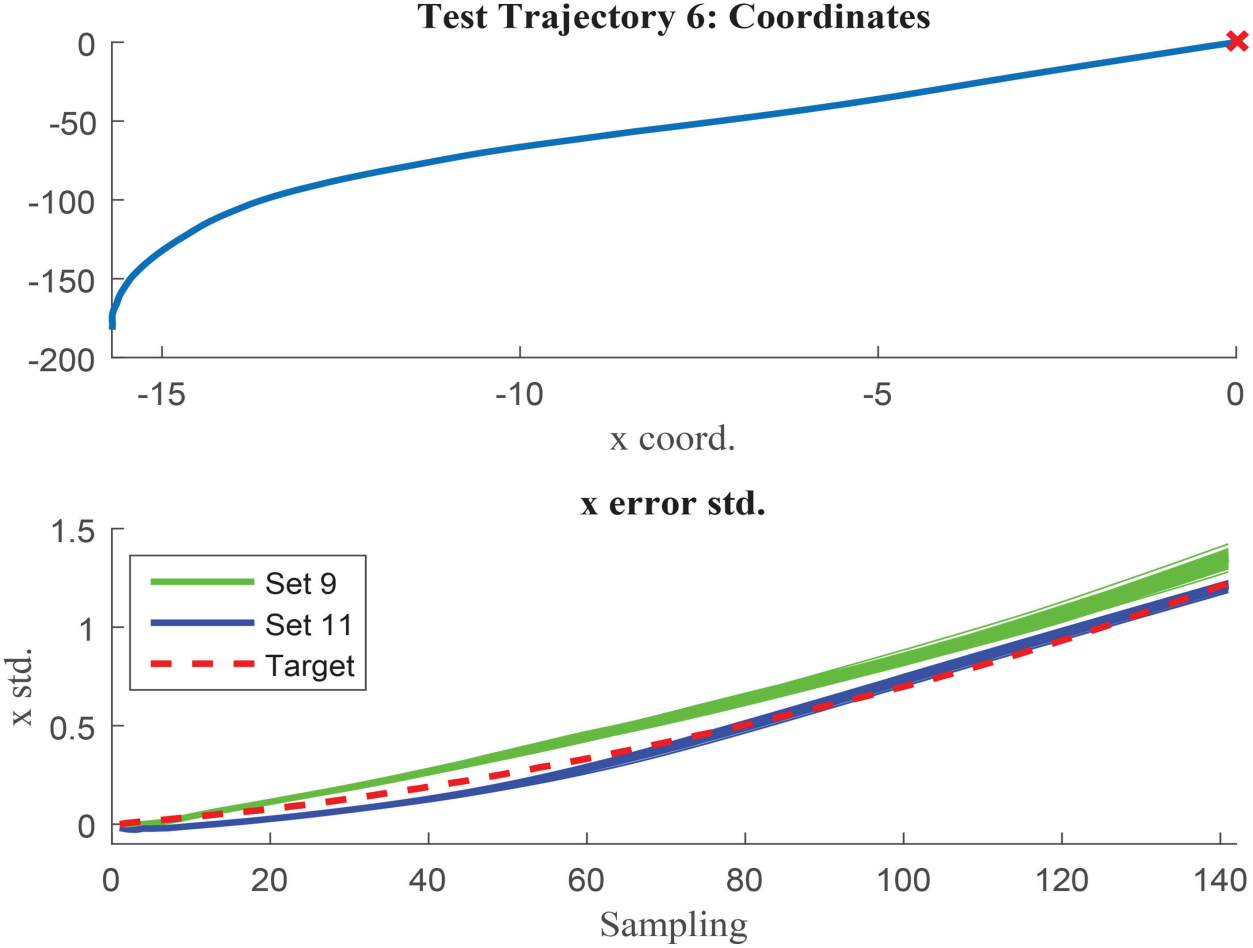
Test trajectory not used in either set 9 or set 11 where set 11 outperforms set 9. Test trajectory 6 comparison of σ_*x*_.

**Figure 15:**
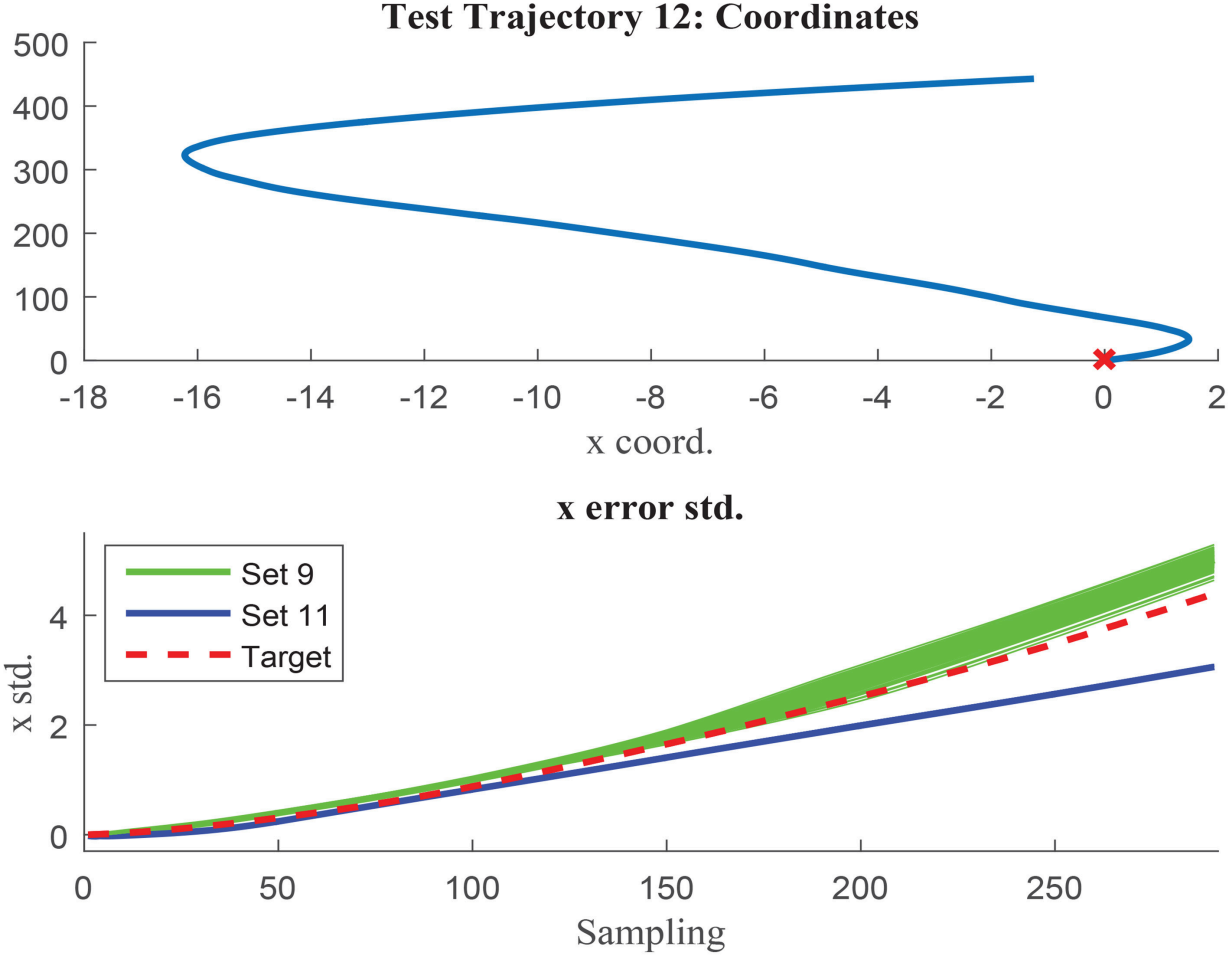
Test trajectory not used in either set 9 or set 11 where set 9 outperforms set 11. Test trajectory 12 comparison of σ_*x*_.

The networks results are visually compared in Figures 14, 15, where test trajectories 6 and 12 are illustrated. None of these trajectories have been used to train any of the networks and therefore, the results can be interpreted as pure testing. The uncertainty estimation in the 1000 MC using the network trained with set 9 is represented in green, whilst the respective results of the network trained with set 11 are illustrated in blue. The target curve is illustrated in red in both cases. Figure 14 shows how set 11 outperforms set 9 prediction results, whilst Figure 15 illustrates the opposite case. Although the results of both networks are rather similar in terms of accuracy, set 11 prediction in the 1000 MC noisy versions of each trajectory are less spread than the equivalent ones from set 9. The larger variability of data used in set 11 seems to have the effect to improve the prediction robustness to noise, and therefore reduce the variability of the prediction when noisy versions of the same trajectory are used. It can be deduced that the consistency of the results is improved when implementing sets with larger variability due to improved noise robustness.

After identifying the most suitable set for training, the optimized number of neurons was investigated. The previous results suggested that the optimal number of neurons is close to 40, probably situated between 40 and 50 neurons. Therefore, further tests were performed using 38, 42, 45, and 47 neurons. The training was maintained until stabilization of the networks performance and allowing generally higher number of epochs for larger sizes. The results verify the a priori hypothesis and situate the best candidates within 38 and 42 neurons. In particular the 42-neurons candidate improves the RMS error in 14 and 18% the 40 and 45-neurons candidates respectively. The cumulative RMS and end errors are also noticeable improved. The RMS error is improved by 0.0296 and 0.0184 and the end error by 0.161 and 0.457, when compared to the 40 and 45-neurons networks respectively. It can be therefore concluded that the optimal network size is 42-neurons hidden layer.

## 7. Conclusions

This paper proposes a strategy for feature uncertainty estimation directly from data without prior knowledge of the sensors characteristics. NNs learning capability of non-linear processes is tested in the particular application of vehicle location through odometry measurements. Both input set and network structure design are based on training and testing results obtained with various neural network candidates. The final results confirm NNs as suitable surrogate modeling technique robust to changes in the testing data, inputs noise and variable case scenarios, provided that the training data captures enough data variability and the network size and structure complexity is able to resemble the process non-linear characteristics.

## Author Contributions

All authors listed have made a substantial, direct and intellectual contribution to the work, and approved it for publication.

### Conflict of Interest Statement

CM was employed by company Porsche Engineering Services GmbH, DC was employed by company Cogsense Technologies Limited. All other authors declare no competing interests.
